# Insights into semi-continuous synthesis of iron oxide nanoparticles (IONPs) via thermal decomposition of iron oleate

**DOI:** 10.1186/s11671-024-04167-6

**Published:** 2025-01-07

**Authors:** Egon Götz Höfgen, Sulalit Bandyopadhyay

**Affiliations:** Particle Engineering Centre, Department of Chemical Engineering, Norwegian University of Science and Technology, Trondheim, 7491 Norway

**Keywords:** Magnetic nanoparticles, Thermal decomposition, Extended LaMer, Semi-continuous, Iron oxide nanoparticle, Iron oleate

## Abstract

**Abstract:**

The increasing demand for magnetic iron oxide nanoparticles (IONPs) in biomedicine necessitates efficient and scalable production methods. Thermal decomposition offers excellent tailoring of the particle properties but its discontinuous batch-operation is restricting scale-up and industrial application. To overcome these challenges, several studies have demonstrated semi-continuous thermal decomposition by slowly injecting the precursor, though only half of them produce magnetite IONPs and even fewer use iron oleate precursors. The available studies are limited, often focusing on single synthesis variables and a comprehensive mapping of the physicochemical properties to reaction conditions is missing. Here we present our investigation of semi-continuous thermal decomposition of iron oleate as a route for the synthesis of magnetic IONPs. We achieved the semi-continuous synthesis of spherical IONPs with properties matching those obtained via the conventional heat-up method. We explored the the effect of multiple synthesis variables, namely addition rate, dwell time, iron oleate amount, oleic acid amount, temperature and consistently report magnetic saturation of our samples. We found that the dwell time seemingly has a stronger effect on particle sizes and magnetic saturation than the addition speed, within moderate addition rates, and further are we the first to report the effect of reaction temperature on semi-continuous synthesis. The iron oleate precursor obtained from salt exchange was employed without pretreatment or dilution thereby facilitating a streamlined synthesis process. An oxidative phase transfer was used to mitigate the key challenge of hydrophobicity of oleate-capped IONPs, enabling their potential use in biomedical applications. Our work advances the understanding of of synthesis-property relationships of IONPs by demonstrating the translation of established synthesis protocols into more efficient and scalable processes through which it provides insights for developing and optimizing future production protocols for various applications.

**Graphical abstract:**

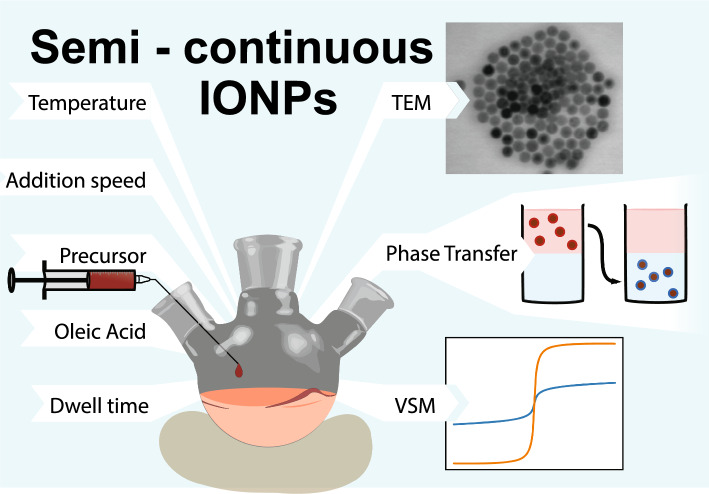

**Supplementary Information:**

The online version contains supplementary material available at 10.1186/s11671-024-04167-6.

## Introduction

Iron oxide nanoparticles (IONPs) are extensively utilized in life sciences and medicine primarily due to their superparamagnetism, which is leveraged for imaging, hyperthermia therapy, sample preparation in diagnostics, and drug delivery among others [1–5]. The growing repertoire of applications necessitates effective production techniques, catering to the specific properties demanded, since, such physicochemical properties, including size, polydispersity, shape, phase and magnetism are controlled by the choice of synthesis route. Hence, it is pivotal to achieve precise control over the synthesis conditions and understand their influence on the final particle properties.

As a result of decades of research, there is an abundance of synthetic approaches for IONPs such as co-precipitation, pyrolysis, thermal decomposition etc., each having its advantages and drawbacks concerning ease of tuning particle properties during synthesis, process complexity and cost, scalability and more [4]. Among these methods, thermal decomposition is widely used due to the clear advantage of precise tailoring of properties by simple synthesis variations. Thermal decomposition involves heating an organometallic iron precursor, predominantly iron oleate (FeOl) [1, 6], to induce thermolysis, releasing reactive iron species into solution, and thereby initiating particle nucleation. A tunable size range of typically 2 to 40 nm [1] in a variety of geometric shapes [1, 7] and structures like single crystals, core-shell etc. of different magnetic properties [8] are accessible by facile adjustments of precursors and/or additives.

Despite its straightforward procedure, the industrial translation of thermal decomposition is primarily hindered by the fact that such processes are commonly conducted as one-pot/batch reactions, where scalability is restricted by mixing, heat and mass transfer [3, 9]. Such drawbacks may be overcome by using continuous processes, which are favoured industrially owing to their ability to achieve consistent quality, scalability, and higher throughput [3, 10, 11]. However, to exploit the advantages of transitioning into a continuous mode of operation (reagent feed and product removal), it is imperative to study how the synthesis conditions in a semi-batch/semi-continuous process (reagent feed alone) affect the physicochemical properties of the resultant IONPs. As an initial approach, a semi-continuous synthesis of IONPs was first realized by Ho et al. by slowly injecting an iron acetylacetonate precursor into the reaction mixture [12]. Vreeland et al. were the first to study thermal decomposition of an iron oleate precursor in semi-continuous mode which catalyzed further research for better mechanistic understanding or scalability of the production of IONPs, via semi-continuous thermal decomposition, including different pathways, precursors, and ferrite materials as shown in the supplementary summary table A1 [13–20].

Firstly, the design space for such semi-continuous thermal decomposition comprises numerous reaction variables (precursor concentration/amount, precursor addition rate, temperature (T), etc.). Literature shows sparse studies with focus on single synthesis variables and inadequate mapping of physicochemical properties to reaction conditions. Two studies demonstrate a linear effect correlating particle volume and amount (n) of iron added (as precursor), with higher precursor quantities yielding bigger IONPs (9 to 35 nm) [16, 21]. Investigation into other synthetic variables remain limited to capping agent concentration or composition of the gas atmosphere [12, 14, 20]. A comprehensive comparative study simultaneously evaluating the influences of multiple variables is lacking. Additionally, if these are reported, magnetic properties are limited to selected experiments, hindering the establishment of correlations between synthesis conditions and properties.

Secondly, what is considered the “iron oleate precursor" is not a defined chemical compound but a complex mixture which is challenging to characterize and very sensitive to preparative variations [6, 22–24]. The unresolved and changing structure makes it highly non-trivial to correlate the FeOl characterization to the IONP properties, in both heat-up [6, 22, 23] and semi-continuous processes [20, 21]. For example, in heat-up synthesis, extended drying at ${110}\,^\circ$C reduces the IONP size [23], whereas multiple-day drying of the iron oleate at room temperature is found to lead to bigger particles [25]. The lack of availability of highly advanced techniques in standard laboratory settings to resolve internal structures of FeOl further amplifies these challenges [26, 27]. while it may be seen as an argument that the synthesis of IONPs is integral to the FeOl characterization.

Third and lastly, the direct biomedical application in aqueous media is hindered by the hydrophobic nature of such oleate-capped IONPs, which can be easily addressed by a phase transfer (PT).

In the present study, we thoroughly characterized the iron oleate precursors produced using salt exchange via infrared spectroscopy (FTIR), thermogravimetric analysis (TGA), and microwave-assisted plasma atomic emission spectroscopy (MP-AES), before employing them for the synthesis of IONPs using both batch and semi-continuous routes. The semi-continuous operation was further studied through a screening of different synthetic conditions, including the temperature effect which has not been reported previously. Seeking to elucidate their effects on the magnetic properties of the IONPs, magnetic characterization has been carried out for all samples. Thereafter, we have rendered our semi-continuously produced IONPs hydrophilic by oxidation of the hydrophobic oleic acid coating. These hydrophilic IONPs are stable in a physiological pH range and can potentially serve as candidates for biomedical applications.

## Results and discussion

### Iron oleate

**Fig. 1:**
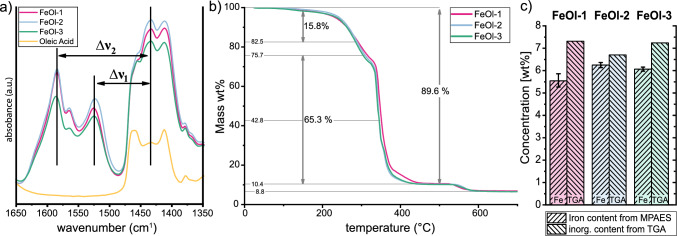
Characterization of three iron oleates (FeOl-1; -2; -3). **a** FTIR-spectra detail of carboxyl stretching vibrations; $\Delta \nu$ distances used to identify the coordination modes $\Delta \nu _1$ 93; 91; 93 $\textrm{cm}^{-1}$- bidentate and $\Delta \nu _2$ = 152; 150; 152  $\textrm{cm}^{-1}$ - bridging; **b** TGA, depicted mass losses as mean of the 3 FeOls; **c** mass based concentration as wt-% of inorganic content through TGA (right bars) and wt-% of iron through MP-AES (left bars, error bars equal standard deviation of 3 digestions)

In contrast to its facile synthesis procedure via salt exchange [28], the characterization of iron oleate (FeOl) is challenging, which hinders the establishment of a correlation between FeOl characterization and resultant IONP properties. Several studies have shown how small variations, eg. of the Fe oxidation state and counter-ions in the iron salt, and reagent ratios, and prominently, the drying/annealing procedures in the preparation of the iron oleate may strongly affect such properties of the IONPs [1, 6, 20, 22–25, 29].

We have synthesized three batches of iron oleate and characterized them using FTIR spectroscopy, TGA, and MP-AES, as depicted in Fig. 1. The characteristic bands observed in the FTIR spectra of all three samples were consistent, albeit slight intensity variations remain post-normalization. These bands align with previously reported FTIR data for similarly prepared iron oleates, as documented in the supplementary information (Figure A1, Table A3 ). Analysis of the carboxyl-stretching vibrations between 1650$\,\textrm{cm}^{-1}$ to 1350 $\textrm{cm}^{-1}$, shown in Fig. 1a, revealed a mixture of bridging and bidentate coordination of iron to the oleate’s carboxyl group, as evidenced by the observed splits in vibration frequencies. To be exact, a split of $\Delta \nu _1={93\,\textrm{cm}^{-1}; 91\,\textrm{cm}^{-1}; \textrm{ and }\ 93}\,\textrm{cm}^{-1}$ indicates bidentate, and $\Delta \nu _2 = {152\,\textrm{cm}^{-1}; 150\,\textrm{cm}^{-1};\ \textrm{and}\ 152\,}\textrm{cm}^{-1}$ bridging for the respective FeOl-1, -2 and -3 in order [22, 23, 25]. The coexistence of both coordination states is consistent with previous reports [6, 22, 23, 25, 30, 31], and together with the absence of notable differences in the full-range FTIR spectra (see SI Figure A1, Table A3 ) it suggests qualitative similarity among the three iron oleate batches produced.

In the thermal decomposition synthesis process, the temperature response of the precursor is of interest, as it is underlying the mechanism of particle formation. In order to study this, TGA was performed on each FeOl sample in a range of 25–${800}\,^\circ$C with a heating rate of 20 K $\hbox {min}^{-1}$. The TGA profiles were almost identical for all three FeOls as shown in Fig. 1b. The main transitions in the mass-temperature plot are given as averages of individual values from each iron oleate given (SI Table A4, Figure A4 ). The first mass loss (17.3 wt%) is in the range ${160}\,^\circ$C until ${287}\,^\circ$C, followed by a small step until ${310}\,^\circ$C, with a total mass loss of 24.3 wt %. Both transitions account for desorption of oleate ligands, as reported in earlier works [22, 30, 32, 33]. However, there is no consensus on the nature of these lost ligands. According to DFT calculations, two of the oleates are bound less strongly, causing earlier desorption in the first step [32]. On the other hand, Solodov and colleagues report an initial 23 wt % loss due to unbound oleic acid present in the iron oleate [30]. In our case, the loss of further oleate ligands continues until ${348}\,^\circ$C and after that, the mass loss is due to evaporation of the organic decomposition products, which ceases at ${500}\,^\circ$C, accounting for a total mass loss of 89.6±0.2 wt % with standard deviation from taking the average of all three FeOls. In the curve’s tail, the mass is reduced by a final 3 wt % through an inorganic phase transition ending at ${600}\,^\circ$C. Using these TGA data, the inorganic residue (T$>{600}\,^\circ$C) is found to be in the range of 6.7–7.3 wt % for the three FeOls studied, represented individually as bars in Fig. 1c. Our results are in agreement with literature reports of 6.0 to 7.42 wt % [22, 30]. For all FeOls, the percentage of inorganic residues is determined to be higher than the content of elemental iron measured through MP-AES after digestion, see Fig. 1c. MP-AES results give an indication of the elemental iron present in the iron oleate, whereas the TGA results also include possible other compounds formed during the measurement process, which account for a maximum difference of 1.8 wt% between the two values. Although nitrogen atmosphere is used in the latter case, residual oxygen from oleic acid could facilitate the formation of iron oxides, thus explaining the different weight percentages found.

The iron concentrations in FeOl is intricately tied to the amount of coordinated and free oleic acid and hydration water present in the mixture, thereby impacting coordination type and strength between the ligands and the iron core. This variable coordination in the FeOl profoundly influences the mechanism of oleate chain dissociation, which is crucial for generating reactive monomers that are essential for IONP nucleation and growth, as well as the total iron amount supplied by the FeOl, which in turn governs properties like the final particle size, etc. [27]. The reported iron concentrations in the literature range from 60 mg $\hbox {g}^{-1}$ to 76 mg $\hbox {g}^{-1}$ [6, 23–26], while our MP-AES analysis (Table 1) reveals iron concentrations in our FeOl samples ranging between 55.5 mg $\hbox {g}^{-1}$; and 62.5mg $\hbox {g}^{-1}$, which may be used to conclude the number of bound oleates and the complex structure.

The iron concentration of FeOl-2 and FeOl-3 exhibit a close resemblance to a triironoxonium core [$$\textrm{Fe}_{3}\textrm{O}]^{n+}$$ comprising nine oleate ligands ($$\hbox {OA}^-$$) alongside some water. Various configurations of this complex have been reported. [6, 24, 26, 27], for example, as [$$\textrm{Fe}_{3}$$O(OA$${^-})_{6}$$] [$$\hbox {OA}^-]\cdot$$(OAH)$$_{2}\cdot$$($$\hbox {H}_{2}$$O)$$_{3}$$ with two unbound oleic acids (OAH) proposed by Chang and Kim et al. The authors have calculated the iron concentration to 60.4 mg $$\hbox {g}^{-1}$$ which they supported by ICP-MS measurements of 59.6 mg $$\hbox {g}^{-1}$$, identical to our FeOl-3’s concentration [26]. Interestingly, FeOl-1 displays the lowest iron concentration, deviating by -5 mg $$\hbox {g}^{-1}$$ to FeOl-2 and 3, possibly attributed to one additional [$$\hbox {OA}^-$$] or potentially 6 water. However, considering the minimal dehydration ($$<{2}\,{wt}\%$$) observed below $${160}\,^\circ$$C in TGA, the significant water content seems improbable, suggesting that FeOl-1 likely contains ten oleates per [Fe$${_3}\textrm{O}]^{n+}$$ center.

The characterizations conducted on our synthesized iron oleates align closely with findings reported in existing literature. The slight deviations observed in FTIR and TGA measurements among the three distinct FeOls remain well within established ranges. As the iron concentration is the only pronounced difference between the FeOls, we proceed our investigation on IONP synthesis, aiming to elucidate its consequential effects on particle formation.

**Table 1:** Measurement from MP-AES with standard deviation of 3 samples, oleic acid (OAH), oleate ($\hbox {OA}^-$). Literature iron concentration calculated based on reported condensed formulas

Sample	Iron concentration $\hbox {mg}_{\textrm{Fe}} \hbox {g}_{\textrm{oleate}}^{-1}$
Iron oleate 1	55.45 +$-$2.80
Iron oleate 2	62.49 +- 1.13
Iron oleate 3	60.68 +- 0.91
Fe($\hbox {OA}^-)_3\cdot$2$\hbox {H}_{2}$O	59.62
Fe($\hbox {OA}^-)_3\cdot$6$\hbox {H}_{2}$O	55.36
$\textrm{Fe}_{3}$O($\hbox {OA}^-)_9$	61.64
[$$\textrm{Fe}_{3}$$O($$\hbox {OA}^-)_6$$] [$$\hbox {OA}^-]\cdot$$(OAH)$$_{2}\cdot$$$$\hbox {H}_{2}$$O [22]	61.19
[$\textrm{Fe}_{3}$O($\hbox {OA}^-)_6$] [$\hbox {OA}^-]\cdot$(OAH)$_{2}\cdot$($\hbox {H}_{2}$O)$_3$ [26]	60.40
[$\textrm{Fe}_{3}$O($\hbox {OA}^-)_6$($\hbox {H}_{2}$O)$_{3}$][$\hbox {OA}^-]\cdot$(OAH)$_{2}\cdot$($\hbox {H}_{2}$O)$_{1.5}$ [24]	59.81
[$\textrm{Fe}_{3}$O($\hbox {OA}^-)_6$($\hbox {H}_{2}$O)$_{3}]^-\mathrm{OA^-}$ [25]	75.85

### Heat-up synthesis

**Fig. 2:**
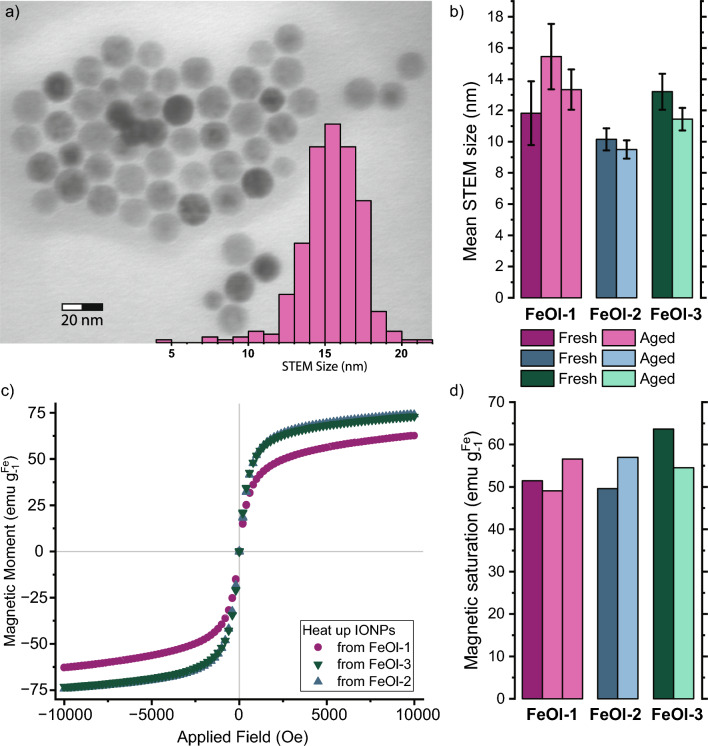
IONPs obtained through heat up method from FeOl-1; -2; -3, fresh and after aging. **a** STEM micrograph and histogram of the first bacth of particles from aged FeOl-1. **c** hysteresis curve of room temperature magnetic response, corrected to iron content of IONPs from fresh FeOls; **b** Mean STEM sizes of repeated and **d** room temperature magnetic saturation of heat-up syntheses of fresh and aged FeOls

The three iron oleates (FeOl-1; -2; -3) were used to produce IONPs following the heat-up method [28]. All of them yield spherical particles, apparent from the Bright-Field TEM micrographs taken on a scanning transmission electron microscope (STEM), of which a representative image being shown in Fig. 2a. The inset histogram shows the size distribution of the IONPs, which is consistent with IONPs previously reported from our labs using the same method [34–36]. Figure 2b and c present the mean STEM sizes and magnetic saturation values of synthesis repeats from the same FeOls freshly prepared and aged. This aged refers to one month of cold storage time for FeOl-1 and 2.5 months for FeOl-2 and FeOl-3, all individual values are given in SI Table A5. The average room temperature magnetic saturation ($M_{sat}$) across all seven samples is ${55\pm 5}\,\text {emu g}_{\textrm{Fe}}^{-1}$ the individual $M_{sat}$ values for each synthesis repeat in Fig. 2d and SI Table A5 indicate no consistent effect from ageing or variations in iron concentration in the oleate. However, the magnetic saturation values are observed to vary proportionally with the sizes of the IONPs, with bigger particles showing slightly higher magnetic saturations [18, 25, 37]. The reporting of magnetic properties in literature varies, some report emu or A $\hbox {m}^2$ per mass of the whole sample, per inorganic/oxide mass, ie., TGA-adjusted, or per specific iron phase or metal content through digestion and element measurements (AAS, ICP-OES, UV/Vis essays, etc.), and sometimes it lacks specification what mass is used as base [6, 14, 18, 33]. Our samples show a low magnetic saturation in the range of 8 emu $\hbox {g}^{-1}$ to 13 emu $\hbox {g}^{-1}$ for oleic acid coated particles, which respectively increase to 50 emu $\hbox {g}_{\textrm{Fe}}^{-1}$ 64 emu $\hbox {g}_{\textrm{Fe}}^{-1}$ after correction from MP-AES for Fe content determination. The ranges measured in our samples with and without correction from MP-AES (SI Table A5 ) are consistent with values reported in prior literature [25, 33, 34, 38].

On average, FeOl-1 batches yield larger particles, ranging from ${15.5 \pm 2.1}$ nm to ${11.8 \pm 2}$ nm, while the smallest particles are produced from FeOl-2, ranging from ${10.2 \pm 0.7}$ nm to ${9.5 \pm 0.6}$ nm. This tendency follows the trend seen for iron concentration observed in MP-AES analysis, giving low $M_{sat}$ values of 55 $\hbox {mg}_{\textrm{Fe}} \hbox {g}_{\textrm{oleate}}^{-1}$ for FeOl-1, 61 $\hbox {mg}_{\textrm{Fe}} \hbox {g}_{\textrm{oleate}}^{-1}$ for FeOl-3, and the highest of 62 $\hbox {mg}_{\textrm{Fe}} \hbox {g}_{\textrm{oleate}}^{-1}$oleate for FeOl-2. X-ray diffraction (XRD) analysis was performed to investigate the iron oxide phase composition of the produced IONPs, as shown in SI Figure A5. The broadening of signals due to the small size restricts to differentiate between the reflections of magnetite and maghemite in powder XRD [14, 25]. All XRD patterns of IONPs from FeOl-2 and FeOl-3 matched to those of magnetite phases. In the diffractograms from IONPs produced from aged FeOl-1 aditionally matched to wüstite phases too, compare SI Figure A5. Partially due to very broad peaks because of small sizes and little sample amounts, but characteristic changes are noticeable in the diffractogram like the (311) magnetite peak around ${34.6}^\circ$ becoming a shoulder to (111) wüstite at ${36.2}^\circ$, which has been reported previously [25]. This appearance of antiferromagnetic wüstite phases provides an explanation to why heat-up IONPs from FeOl-1 have the lowest magnetisation of the three.

As has been reported in prior literature [23, 28], size of synthesized IONPs can be tuned between 5 and 40 nm by varying the synthesis conditions, specifically the amount of iron oleate and its treatment. Notably, ageing of FeOl-2 and -3 resulted in a slight size reduction, whereas for IONPs synthesized from FeOl-1, ageing seemingly had the opposite effect. However, these tendencies lack statistical significance when considering the standard deviations observed between IONP syntheses and between different FeOls. Unlike our findings, Balakrishnan et al. reported a size increase with ageing, doubling the particle size to a final of 13.1 nm, with a linear progression noted between 5 and 10 days [25]. In contrast to our FeOl synthesis method, where hexane is removed in vacuo, their ageing procedure involves open drying at ${30}\,^\circ$C without prior removal of hexane. Further, our FeOls were stored in airtight glass vials at ${4}\,^\circ$C which were only opened at room temperature to take aliquots needed for synthesis and characterization. Another previous report using the same preparation of FeOl, as in our study, observed significant size reduction of the particles induced by extended annealing at ${110}\,^\circ$C [23]. In our measurements of magnetic saturation, we observed no effect for particles obtained from 2.5 months aged FeOls.

In an attempt to understand the ageing, FTIR analysis was carried out for the aged FeOls, shown in SI Figure A2. Comparing the FTIR spectrum of fresh and aged FeOl, the vibrations of the aliphatic OA backbone in the band of 2800–3000 $\textrm{cm}^{-1}$ were found to remain unchanged; hence, they were also used to normalize the spectra of the aged FeOls. In the fingerprint region, the absorbance is seen to increase slightly, with exception for the peak at 1711 $\textrm{cm}^{-1}$, which reduces in intensity, depicted in SI Figure A3. This mode of $\nu _{s}$(C=O) is indicative of free or unbound oleic acid [6, 30, 39]. The reduced signal means that this carbonyl oxygen enters the coordination of the iron complex, since during closed storage there are no alternative explanations for the loss of a carbonyl group. From comparison of the iron content in FeOls (Table 1), we believe in a structure of 9 or 10 oleate per triironoxonium core. However, there seems to be rearrangement between associated/free and bound/twice coordinated oleate, which cannot be inferred from MP-AES data. As the coordination mode of C=O reduces, the carboxyl coordination modes (${1645}\,\hbox {cm}^{-1}\ \textrm{to}\ {1390}\,\hbox {cm}^{-1}$) increase in intensity accordingly, without changing peak shape. We note that the distance $\Delta \nu _1$ (bidentate) reduces slightly by 2 $\textrm{cm}^{-1}$, whereas $\Delta \nu _2$ (bridging) increases by ${2}\,\hbox {cm}^{-1}\ \textrm{to}\ {4}\,\hbox {cm}^{-1}$ with ageing for all three FeOls. This observed shift is in line with what has been reported previously. [23, 31] Stronger coordination, i.e., bidentate and bridging, between iron and oleate carboxylates requires more energy to separate. Consequently, the nucleation temperature is increased [23, 31], which in turn leads to smaller particles [23]. This nominal tendency for smaller particles was observed in our produced particles, however, as it is within the inter batch standard deviation and the diameters are consistently in the range of 10–15 nm with unaffected magnetic saturation of ${55 \pm 5}\ \hbox {emu g}_{\textrm{Fe}}^{-1}$. We proceed under the assumption that the semi-continuous synthesis and parameter study performed throughout this ageing period plausibly is unaffected by ageing.

### Semi-continuous

**Fig. 3:**
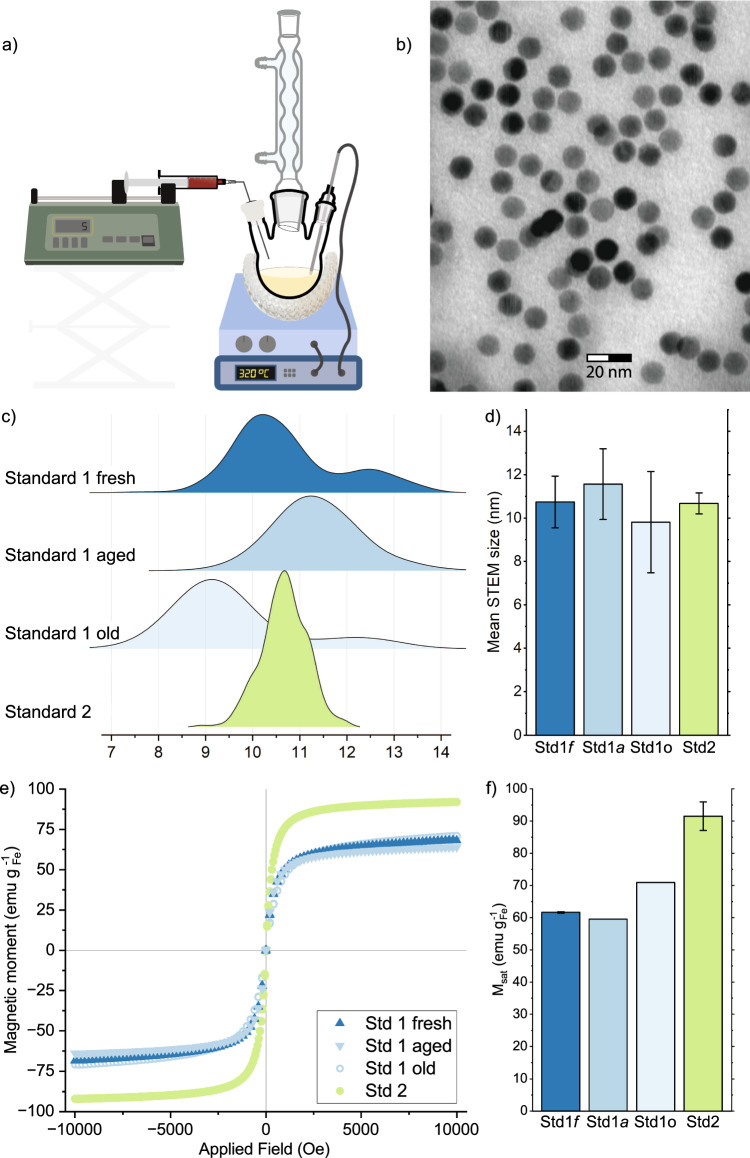
Summary of semi-continuous standard reactions **a** Schematic setup and characterization of IONPs produced **b**–**d**. STEM characterization **b** STEM image of Standard 2 from FeOl-3, **c** size distribution, **d** mean STEM sizes corresponding to **b**, **e** magnetisation curve of room temperature magnetic response, corrected to iron content and **f** magnetic saturation. Std 1 after different times of ageing FeOl-2, aged=2.5 months, old = 1 year

To realise the semi-continuous approach of synthesis of IONPs, the as-prepared FeOl precursor was injected through a syringe, using a syringe pump, into the hot octadecene-oleic acid mixture, as shown in Fig. 3a. Drawing from insights gained during preliminary experiments, a specific set of reaction conditions was selected to produce IONPs of sizes and magnetic saturations comparable to those achieved via heat-up synthesis (batch process). Termed the “standard" semi-continuous synthesis, the selected reaction conditions were set to: 900 mg iron oleate, 1.5 eq. (527 ${\upmu \hbox {L}}$) oleic acid, 25 mL octadecene, pump set to 5 $\hbox {mLh}^{-1}$ addition speed, conducted at ${320}\,^\circ$C with a dwell time (*t*) of 30 min. Utilizing this standard synthesis, spherical particles were obtained as shown in Fig. 3c, characterized by a narrow size distribution illustrated in Fig. 3b. FeOl-2 was used for reactions named standard 1 (Std 1), and FeOl-3 yields standard 2 (Std 2) respectively. STEM sizes measured for standard 1 (${10.7 \pm 1.2}$ nm) and standard 2 (${10.7 \pm 0.5}$ nm) exhibit such minimal divergence that they can be considered equivalent. The STEM sizes of 10 nm to 11 nm lie in between the batch sizes of ${9.8 \pm 0.3}$ m and ${12.3\pm 0.9}$ nm of FeOl-2 and FeOl-3 respectively, and fall in the lower range for previously reported semi-continuous syntheses [15, 16, 21].

While size, shape, and superparamagnetic behaviour (Fig. 3e) are consistent in the two standard reactions produced from the two fresh FeOl batches, the magnetic saturations differ. The slight difference seen in heat-up that FeOl-3 produced particles with higher magnetic saturation than FeOl-2, is even more pronounced in semi-continuous operation. $M_{sat}$ of standard 1 of fresh FeOl-2 (Std 1*f*) and aged are approximately at 60 $\hbox {emu g}_{\textrm{Fe}}^{-1}$, whereas standard 2 (FeOl-3) is 30 $\hbox {emu g}_{\textrm{Fe}}^{-1}$ higher, but still within the range of magnetic saturation values reported for IONPs in prior semi-continuous studies [14, 21]. Since $M_{sat}$(Std 2) is exceeding all other samples by at least 10 $\hbox {emu g}_{\textrm{Fe}}^{-1}$ we decided to treat it as an experimental outlier and exclude it from the following discussion of the effects of the reaction variables.

Standard 1 was repeated after 2.5 months (Std 1 aged or Std1*a*) and after one year of FeOl storage (Std 1$\circ$) to test for repeatability and ageing effects of the FeOl. The magnetic saturation remained constant after 2.5 months (60 $\hbox {emu g}_{\textrm{Fe}}^{-1}$, Fig. 3e and the STEM size changed slightly to ${11.5\pm 1.6}$ nm (Std 1*a*) and ${9.8\pm 2.3}$ nm after one year (Std 1$\circ$) with increasing standard deviation. From the size distributions in Fig. 3c it is apparent that the slight bimodality of Std 1*f* has disappeared and centered into a monomodal but broadened distribution in Std 1*a*. As the absolute size difference is within the standard deviation, we interpret it as unchanged.

Powder XRD confirmed magnetite as phase for all standard semi-continuous reactions with no evidence for wüstite, see SI Figure A6, as observed in literature of semi-continuous synthesis [14, 21].

#### Screening of reaction variables

**Fig. 4:**
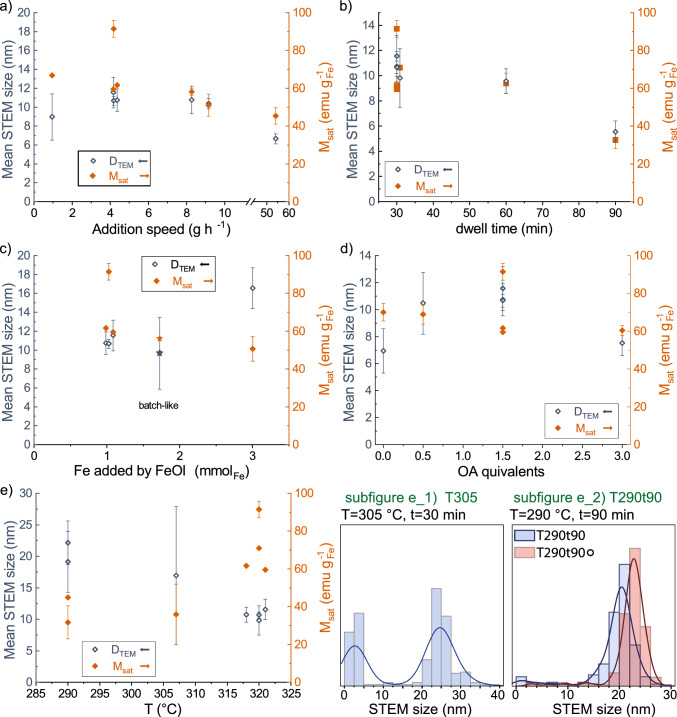
Effect of changed synthesis variables **a** addition speed, **b** dwell time, **c** amount of iron added through the FeOl precursor, **d** oleic acid (OA) amount as equivalents of *n*(FeOl), and **e** final reaction temperature on the resultant IONPs’ properties. Left axis, hollow symbols STEM size; right axis, full orange symbols magnetic saturation corrected to iron content. Subfigures e_1), e_2) show the size distribution of the low-temperature reactions, e_1) synthesized with 30 min dwell at ${305}\,^\circ$C, e_2) 90 min dwell at ${290}\,^\circ$C of fresh FeOl-2 and 1 year aged T290t90$\circ$

We conducted multiple screening experiments to explore the effects of different reaction conditions on the physicochemical properties of the particles synthesized in a semi-continuous process. Each experiment involved changing one of the five reaction variables (*n*(FeOl), $t,\ T$, oleic acid amounts as equivalents to *n*(FeOl) (eq.(OA)), addition speed) while keeping the others constant at the same settings as those of the standard semi-continuous. (See Table 2 in Sect. 4 Methods for the complete list of experiments performed.) For two specific experiments, two variables were changed simultaneously. For the first one, at the lowest temperature of $T={290}^\circ$ the dwell time was increased to 90 min, while for the second one, the precursor amount and dwell time were increased to match the heat-up conditions. From this heat-up like semi-continuous reaction, the IONP’s size (${9.7\pm 3.8}$ nm) is the same as heat-up from the same FeOl-2 of ${10.2 \pm 0.7}$ nm and ${9.5 \pm 0.6}$ nm. However, few non-spherical particles of up to 50 nm could be noticed in the semi-continuous sample, resulting in a high standard deviation of the mean STEM size. The observed effects of the studied reaction conditions on the physicochemical properties of the IONPs are presented in Fig. 4, where the left axis represents average STEM sizes and the right axis represents the magnetic saturation values of the IONPs, values given SI Table A6.

##### Effect of addition speed

During experimental work the addition speed was set on the syringe pump in $\hbox {mL h}^{-1}$ and afterwards corrected by the exact mass of FeOl dispensed and the time required, so the addition speed thereafter is given in $\hbox {g}$ $\hbox {h}^{-1}$. Doubling the addition speed from 4 $\hbox {g h}^{-1}$ in the standard to 8 $\hbox {g h}^{-1}$ has no significant effect on the average size or size distribution of the IONPs, as presented in Fig. 4a. If the addition rate of the precursor is higher than its consumption by growth, it would cause a supersaturation buildup and result in the nucleation of a second population when the minimal nucleation concentration is overcome. The observed monomodal nature of the size distribution plot rules out the possibility of nucleation of new particles. The argument that the total precursor amount added is not sufficient for multiple nucleation events can be rebutted by the bimodal population observed under different conditions, eg. Fig.  4 subfig e_1). Opposite to the unchanged IONP size, is the magnetic saturation slightly lower at faster additions. Plausibly, a faster precursor addition could sustain a faster particle growth, which more easily generates crystal defects adversely affecting the magnetic moment. To raise the addition speed above 8 $\hbox {g h}^{-1}$, a hot injection was performed by manually injecting the FeOl. The resultant particles are even smaller (${6.7\pm 0.5}$ nm) than at $4\ \textrm{g h}^{-1}$ and 8 g $\hbox {h}^{-1}$. A plausible explanation for this comes from classical crystallization theory - as the faster buildup of supersaturation causes a higher overshoot above the minimal nucleation concentration, more nuclei form. As more nuclei grow from the same precursor amount, the particles grow to a smaller final size. The smaller size is reflected in further reduced magnetic saturation compared to standard and 8 $\hbox {g h}^{-1}$ reactions. Lastly, a low addition speed of 0.9 $\hbox {g h}^{-1}$ was tested, resulting in IONPs with smaller size ${8.9\pm 2.8}$ nm and higher polydispersity (27%), compared to the standard. To be attributed to a changed reaction variable, a size difference should be greater than ${\pm 1.6}$ nm, as this is the maximum standard deviation observed within our repeated syntheses in semi-continuous and heat-up reactions. In this light, changing the addition speed of the syringe pump, ie., excluding the manual hot injection, has no definite effect on the mean STEM size of the particles. In an ideal case, the constant addition of precursor suppresses Ostwald ripening as the cause of polydispersity [13]. Such slow growth would further yield uniformly bigger particles. The slowest injection rate lies outside this desired range, evident from its big standard deviation and small particle size.

When the addition rate was decreased to 0.9 $\hbox {g h}^{-1}$, the injection time increased to approximately one hour, resulting in an increased total reaction time of 90 min (including 30 min dwell time after injection). In the standard reaction, the precursor was added at 4 $\hbox {g h}^{-1}$ equal to 13 min injection, plus 30 min dwell time. Doubling the dwell time to 60 min at standard addition rate has no observable effect on STEM sizes, as they stay in the same range as for semi-continuous standard and heat-up reactions, see Fig. 4b. Per contra, for 90 min dwell time, the absolute STEM size is noticeably reduced to ${5.7\pm 0.8}$ nm, with few bigger particles but with a lower polydispersity compared to the slow injection case. Seemingly, the particle size exhibits a stronger correlation to dwell time (Fig. 4b) or total reaction time (SI Figure A7 ) than to the addition speed.

In the context of laboratory experiments, but even more for commercial production, a balance must be found between providing a controlled, reproducible injection and the total time required for the injection and reaction. Continuing from the effects discussed above, the chosen standard addition rate (4 $\hbox {g h}^{-1}$) presents itself as a good compromise, providing both.

##### Effect of Fe-precursor amount

Next, the effect of changed precursor amounts was studied, and the observations are summarized in Fig. 4c. As the two used FeOls slightly deviate in their respective iron concentrations (Table 1), the amount of added precursor was adjusted to reflect the amount of iron provided for the thermal decomposition process. An increased precursor amount yields bigger particles (${16.4\pm 2.2}$ nm), in accordance with literature but in a less pronounced effect. [16, 18, 21] Likely the dwell time of 30 min used in our experiments is too short to effectively consume the additional precursor provided to grow into substantially bigger particles, consequently, there is a higher ratio of unreacted precursor left. This is supported by the observation that drying these particles yields a much more ferrofluid-like substance. At the intermediate point (star symbols in Fig. 4c), the precursor amount was increased to match the heat-up conditions, but this synthesis included prolonging the dwell time to 45 min as well. Its $M_{sat}$ is approximately constant compared to standard, whereas the size dispersity increases, as discussed above.

##### Effect of reaction temperature

At a reduced reaction temperature of ${305}\,^\circ$C (T305), the size distribution splits into two populations of big ones >20 nm and very small particles <5 nm, see subfigure e_1) Fig. 4, resulting in a drastic increase in standard deviation of the average STEM size.

The appearance of a secondary population is indicative of repeated nucleation events [13, 15]. Precondition for second nucleation is the repeated exceedance of the minimal nucleation concentration caused when the precursor addition is faster than its consumption by growth. For example, the lower growth rate of bigger particles resulted in secondary nucleation in a semi-continuous Co-ferrite system when the NP’s grew bigger than 18 nm [15]. In our experiment, the growth rate was reduced by lowered temperature, as evidenced by the bimodal size distribution at ${305}\,^\circ$C. The very small size of the secondary particles indicates that the second nucleation appeared close to the end of the injection, reducing the time and precursor available for the new particles to grow. Reported heat-up experiments and our own TG analysis of the FeOls indicated that it might be possible to further reduce the reaction temperature [27, 40]. However, preliminary experiments showed that lowering the temperature below ${305}\,^\circ$C in semi-continuous reactions would not yield obtainable particles within 30 min, thus the dwell time was increased to 90 min. IONPs produced under these conditions of ${290}^\circ$C, 90 min dwell time (T290t90) show a mean STEM size of ${19.1\pm 4.8}$ nm. This experiment was reapeated with one year aged FeOl (same as in Std 1$\circ$), yielding particles of ${22.1\pm 3.4}$ nm. The presence of few smaller particles is the reason for the fronting observed in the particle size distributions (Fig. 4 subfig.e_2)), resulting in a considerable standard deviations. Here, the smaller particles do not make up a distinct second population, in contrast to the experiment at ${305}\,^\circ$C. The absence of a second population at ${290}\,^\circ$C could be explained through the prolonged dwell time not only allowing the particles to form but further undergoing size-focusing and ripening, dissolving the secondary small population. Alternatively, the whole FeOl decomposition at ${290}\,^\circ$C is slowed down to such an extent that there is no secondary nucleation and the observed small particles are remnants of incomplete size-focusing.

The second population of small particles at ${305}\,^\circ$C could potentially be the cause for the reduced magnetic saturation of the whole sample, as very smaller particles have a lower magnetic moment [18, 25, 37]. However, the ${290}\,^\circ$C sample has much fewer tiny particles, but the magnetic saturation is equally low for both cases, with ${32\pm 9}\,\hbox {emu g}_{Fe}^{-1}$ at ${290}\,^\circ$C and ${36\pm 16}\,\hbox {emu g}_{Fe}^{-1}$ at ${305}\,^\circ$C, which cannot solely be explained by the size-$M_{sat}$-effect. The thermal decomposition mechanism of FeOl involves a complex interplay of pathways and reactive species, involving not only the precursor complex but also the simultaneous decomposing of oleic acid and octadecene in a delicate redox balance [24, 26, 30]. An excess of highly reactive species like radicals can lead to over-reduction, reducing more $\textrm{Fe}^{3+}$ than the required ratio necessary for the formation of the desired magnetite phase. The resultant $\textrm{Fe}^{2+}$ excess leads to crystal defects and the formation of undesired oxide phases, ie., antiferromagnetic wüstite FeO, [24] as detected in our XRD patterns (SI Figure A9 ). Apparently, the milder reaction conditions at reduced reaction temperatures were not sufficient to mitigate the over-reduction. An additional reason for wüstite formation and low magnetism is oxygen insufficiency [6, 14, 24]. Unni et al. found that, in fact, oxygen is the limiting factor in growing single crystalline magnetite nanoparticles with high magnetic saturation in heat-up and semi-continuous reactions [14]. During extended reactions, such as the 90 min dwell times at ${290}\,^\circ$C and ${320}\,^\circ$C, the lack of oxygen could become even more pronounced. This leads to the formation of particles of non-magnetic phases or polycrystalline particles with a consequently reduced overall magnetic moment [14, 16]. These particles are still subject to ripening and size-focusing effects, resulting in monodisperse size distributions. In the lower temperature cases, the magnetisation curve is still characteristic of superparamagnetism without hysteresis, however, the shape is distinctively more rounded than others and does not saturate at the maximum field of 10 kOe (SI Figure A8 ). This rounding of the magnetisation curve is another indication of challenges in crystallinity and phase purity [6, 14, 37], but can be caused by size effects as well [18, 25, 37]. Since smaller particles result in more curve rounding, opposite to the big sizes and size distribution the lower temperature samples, we favour the interpretation of the rounded magnetisation curve as a sign of phase differences, supported by our XRD phase analysis. Additionally, larger particles, such as in the ${290}\,^\circ$C samples, are more affected by wüstite impurities. This relationship is directly linked to the particle size, as cores of these larger particles are more effectively shielded from oxidation, which was also observed in the literature where bigger particles show stronger wüstite reflections and flatter magnetisation curves [22, 25]. A detailed look at the XRD diffractograms (shown SI Figure A9 ) show an appearance of more and more wüstite reflections with reduced temperatures. For example, the (400) and (440) reflexes of magnetite at ${43}\,^\circ$ and ${62}\,^\circ$ from Std 1$\circ$ are (partially) replaced by (200) and (220) of wüstite. Further is the (311) of magnetite ${34.6}\,^\circ$C reduced to a shoulder of the (111) wüstite reflection at ${36.2}\,^\circ$. A repeat of T290t90 (marked $\circ$) showed reproduced the discussed effects. Further indications of crystallographic defects, which have negative effect on saturation magnetisation, are observations of inhomogeneous contrast within particles including, potentially cores in STEM images, SI Figure A10.

##### Discussion

Semi-continuous reaction in lean octadecene without additional oleic acid resulted in very small, roughly spherical particles of varying sizes with a mean of ${7\pm 1.6}$ nm, but the particles formed stable aggregates of 50 nm to micrometer size. The aggregates and rough shapes can be a result of the absence of the oleic acid’s stabilizing effect [23, 26, 27]. Using half an equivalent of oleic acid is sufficient to produce single particles of the same size as the standard again, although with much bigger polydispersity. Big excess oleic acid of 3 equivalents, double the amount as in standard, reduces the size (${7.5\pm 0.9}$ nm) compared to the standard. This highlights the important role of oleic acid in nucleation and growth of IONPs [23, 26, 27, 32, 39]. The chosen standard conditions are seemingly an optimum for bigger particles with narrow size distribution, while there was no noticeable effect of oleic acid content on magnetic saturation.

As presented in the SI summary Table A1 are the STEM sizes of the particles in our study are in good agreement with Ferrite-NPs in literature. Notably, Vreeland et al. and Unni et al. are the only reports semi-continuously producing magnetite NPs from the classic thermal decomposition of iron oleates, whereas in conditions different to ours. While their studies report magnetisation values consistent with our findings, their particles sizes were twice that of our standard samples, measuring 22.7 nm with a magnetization of 97 $\hbox {emu g}_{Fe}^{-1}$ [21] and 21 nm with 102 $\hbox {emu g}_{Fe}^{-1}$ [14] (reported as emu g$_{\textrm{Fe}_{3}\textrm{O}_{4}}^{-1}$ and converted by us to emu $\hbox {g}_{\textrm{Fe}}^{-1}$). In the latter case by Unni et al., the high magnetization values were only achievable *via* the controlled addition of oxygen. For IONPs from the same semi-continuous reaction without oxygen addition exhibited a magnetic saturation as low as 23 $\hbox {emu g}_{Fe}^{-1}$ at equal size of 21 nm [14]. We did not investigate this variable; nevertheless are the characteristics of the gas atmosphere, like composition or flow rate [14, 15], important factors in semi-continuous reactions and should be considered especially for scale-up and industrial realisation. An alternative approach to address the challenge of over reduction in future work would be non-aqueous redox phase tuning, where by tailored addition of oxidising reagents the oxide phases can be fine tuned [31, 41]. Mixing or substituting octadecene with dibenzylether or squalene has shown promising results in heat-up synthesis to produce phase-pure single-crystalline magnetite nanoparticles [31, 41]. Adding auxiliary compounds is probably more acceptable than molecular oxygen in a safety-focused industry.

### Phase transfer

**Fig. 5:**
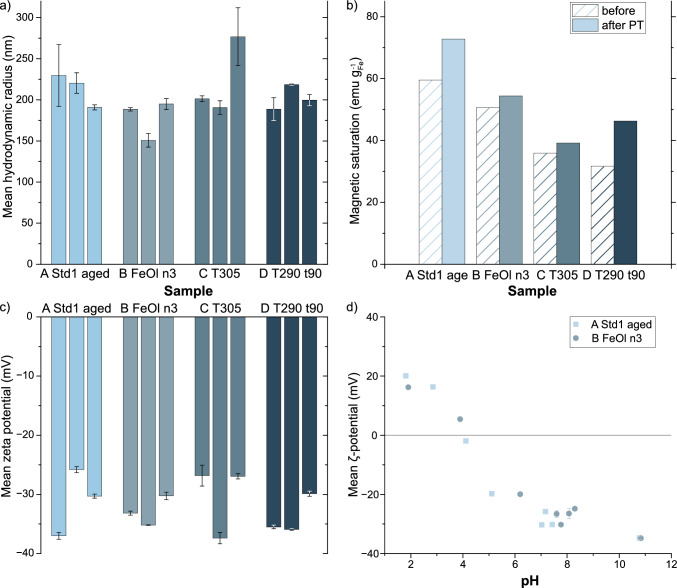
Characterization of IONPs produced by different parameters in semi-continuous **A**–**E** then phase transferred as triplet repeats. DLS characterization at neutral pH to determine **a** mean hydrodynamic radius and c) $\zeta$-potential. **d** $\zeta$ vs. (pH) -curve, **b** magnetic saturation of the IONPs (combined triplet repeats) as syntheised with OA (striped) and after phase transfer (PT) (solid), corrected to iron content

Four samples from the semi-continuous screening study were selected to reflect a range of different particle properties. These hydrophobic particles were rendered hydrophilic by oxidizing the oleic acid with a mix of permanganate and periodate. [42] The phase transferred particles displayed such robust colloidal stability in water that a pH reduction was necessary for effective particle retrieval via centrifugation (20,000 g) at the last washing step. The hydrodynamic radius and surface charge of the obtained particles were characterized using dynamic light scattering (DLS) and electrophoretic measurements, presented in Fig. 5. Electrophoretic measurements showed that the particles exhibit a high negative $\zeta$-potential of ${-32\pm 4}$ mV (Fig. 5c), which is in line with previous reports [42]. The average hydrodynamic radius of the particles is found around 215 nm, which indicates aggregation rather than a dispersion of individual particles despite the high surface charge, which should prevent aggregation. Imaging the transferred particles in STEM reveals that their size is unchanged by phase transfer, but it is apparent that there is a mix of single particles, clusters of few particles, and aggregates up to micrometer size, see SI Figure A12. Consequently, the size observed in STEM before phase transfer is not reflected in the hydrodynamic size post-phase transfer. The ratio of single particles to aggregates varies between samples. The micrographs further suggest that the aggregates share a common or fused coating, SI Figure A12. This common coating and the presence of aggregates are indicative of incomplete oxidation. Suboptimal conditions during phase transfer lead to partial oxidation of the oleic acid coating. The partially oxidized particles probably orient their hydrophobic sides towards each other, forming aggregates with hydrophobic centers. The aggregate’s shared surface is accessible for oxidation, providing the surface charge and hydrophilic properties observed. During the different steps of phase transfer, sonication was employed to facilitate dispersion of formed aggregates, but the aggregates persist. The postulated phase transfer mechanism suggests that PVP facilitates the oxidation by penetrating between oleate molecules [42]. Subsequently, these created fissures provide attack points for the oxidation solution [42]. Following this proposed mechanism, the incomplete oxidation and aggregation may potentially be reduced by adjusting the PVP concentration. After the oxidation step, centrifugation showed to be superior in collecting the particles compared to magnetic separation, as it gives access to particles in the organic phase and at the interface. Although these particles remaining in the organic phase likely experienced incomplete oxidation, magnetic separation might be a suitable approach to selectively collecting fully oxidized single particles. The iron concentration of the dried phase transferred particles (triplets combined for drying) was determined through digestion and MP-AES analysis. This iron content in turn can provide a rough estimate of the organic content in the sample, assuming (i) the inorganic phase as $\textrm{Fe}_3\textrm{O}_4$ as the iron oxide phase with the highest oxygen content and (ii) all remaining mass loss equals the organic content. The organic contents estimated in this way range from 0 % in sample A to 50 % for sample C, see SI Table A7. The organic content is probably underestimated by assuming pure magnetite, as 0 % in sample A is rather unlikely, nevertheless, the indication of successful phase transfer is supported as sample A has mostly single particles in STEM, SI Figure A12-A. On the other hand, sample C and D have a high estimated organic content of ca. 50 % and 45 %, respectively. These findings correspond with the synthesis history of C and D, given that the particles were synthesized at lower temperatures, potentially leading to incomplete FeOl decomposition and leaving residual organic material in the samples. Consequently, this propagates as an imbalance in reactants during oxidation and may have resulted in incomplete phase transfer and high organic content, corroborated by the STEM images where we observe an abundance of aggregates. These aggregates are embedded in a common coating, believed to consist of oleic acid or its partially oxidized derivatives, which surrounds the particles to such an extent that even the smaller particles observed in low-temperature samples are fully encapsulated within the coating, SI Figure A12 subfig.D2).

After phase transfer, the particles typically show an increased magnetic saturation, the strongest effect seen for samples A and D which increased approximately 14 emu $\hbox {g}_{\textrm{Fe}}^{-1}$ to 73 emu $\hbox {g}_{\textrm{Fe}}^{-1}$ and 46 emu $\hbox {g}_{\textrm{Fe}}^{-1}$ respectively, details in SI Table A7. Two explanations could plausibly account for this observation. Firstly, aside from the coordination type, the chain length of the ligand has a known effect on magnetization [43]. Hence, modifying the oleate coating by cleaving the oleic acid or reducing its coating density could reduce the magnetic dead layer on the particles’ surface [43]. Secondly, the highly oxidative environment during the phase transfer might have assisted the oxidation of wüstite and other non-magnetic phases in the particles or have reduced the magnetic dead layer and thereby raised their magnetic saturation. XRD analysis performed on sample A match magnetite as dominant iron oxide phase and appears unaffected by the phase transfer (see SI Figure A11). When first reporting this phase transfer protocol, the magnetic saturation was almost unchanged by the phase transfer, ie., from 35 to 39 emu $\hbox {g}_{\textrm{Fe}}^{-1}$ [42], which aligns to our observations in samples B-D. The stability of the phase transferred particles at different pH levels was investigated. The results depicted in Fig. 5d show that the particles remain stable at neutral to alkaline conditions, but below pH 5, the particles start aggregating significantly. The isoelectronic point is determined to be around pH 4.3, and further pH reduction stabilizes the surface charge at approximately +20 mV. However, despite this stabilized charge, the formed aggregates fail to redisperse. A previous report has found no charge of the particles at pH 5 and 3 [42].

To conclude, this demonstrates the applicability of the phase transfer method to semi-continuously produced IONPs. However, due to limited amount of samples the phase transfer protocol was not further optimised as it presumably would need to be adjusted to the properties of each screening sample, as outlined in the preceding paragraph.

## Conclusion

In summary, our investigation into semi-continuous thermal decomposition of iron oleate has yielded valuable insights into understanding the relationship between synthesis variables and particle properties. Through successful implementation of a salt exchange method for synthesis and bypassing pretreatment or dilution of the iron oleate precursor, we realized semi-continuous synthesis of spherical magnetite IONPs, with properties of the resultant IONPs matching to those obtained via the heat-up method. The challenges encountered working with iron oleate, the preferred precursor for thermal decomposition, persist in semi-continuous synthesis. Such challenges arise from FeOl being a non-stoichiometric complex mixture with susceptibility to retain water, oleic acid, and other reaction by-products while resisting purification and thorough characterization of the composition [6, 21, 22]. Instability and small variations between FeOl batches, which are difficult to detect and relate to synthetic outcomes, carry on as minor changes in the nucleation process that cause significant differences in the properties of the final particles. For example, the FTIR spectrum of FeOl-3 shows the strongest free carbonyl peak at 1711 $\textrm{cm}^{-1}$ (SI Figure A3), which is indicative of the amount of unbound oleic acid [6, 39]. However, this is not reflected in the other characterization results from TGA and MP-AES and synthesis of IONPs, in which case FeOl-1 shows the biggest deviation. Nevertheless, all three FeOls produce IONPs of the same quality. Based on our experiences throughout this study, we emphasise that FeOl is notoriously challenging material for laboratory work in direct alignment with previous reports [6, 21]. Moving forward in efforts in up-scaling and commercial application, one can recommend an alternative precursor or different FeOl preparations [6] to remove the high potential of variability and ensure reproducibility.

Our systematic exploration of synthesis variables has uncovered correlations in responses to changed dwell times, temperature, oleic acid, and precursor amounts. Reducing the reaction temperature from ${320}\,^\circ$C during the semi-continuous synthesis has significant effects on the IONPs’ properties. At ${305}\,^\circ$C, a bimodal size distribution emerges, indicating a secondary nucleation event. However, at ${290}\,^\circ$C, extended dwell time allows particles to form and undergo size-focusing, resulting in larger particles without a distinct second population. On the downside, at the trade-off of forming wüstite phases instead of desired magnetite phase which results in rounded magnetisation curves and reduced $M_{sat}$, possibly due to problems arising from oxygen availability or redox imbalance [14, 25, 31, 41], which could be addressed in further studies. Increasing precursor amounts led to larger IONPs ${16.4 \pm 2.2}$ nm, consistent with existing literature [16, 18, 21], but dwell time limitations prevent substantial growth. While oleic acid affects nucleation and growth, the chosen standard conditions produce optimum particle size and narrow size distribution, whereas the magnetic saturation remains unaffected by oleic acid content. Collectively, our findings showcase the nuanced effects changed reaction variables can have on particle size, size distribution and magnetic properties and therefore, provide valuable insights for future studies. Further, the oxidative phase transfer performed on the semi-continuously produced hydrophobic IONPs provides negative surface charges and colloidal stability in water, but incomplete oxidation leads to aggregates around 0.5 ${\upmu \hbox {m}}$ with shared coatings. Thorough characterization and phase transfer helped to address key challenges associated with iron oleate-based IONPs, paving the way for their potential use in biomedical applications. This work progresses our understanding of synthesis-property relationships but also demonstrates the feasibility of translating established synthesis protocols to more efficient and scalable processes.

## Methods

### Synthesis

*Iron oleate synthesis* Three batches of iron oleate (FeOl-1, -2 and -3) were synthesized by the same method from salt exchange with sodium oleate as reported previously [28]. Sodium oleate (0.15 mol = 45.6 g) was suspended in 175 mL *n*-hexane in a round bottom flask, then $\hbox {FeCl}_3\cdot$6$\hbox {H}_2$O (0.05 mol = 13.515 g) dissolved in 62.5mL MQ-water, as well as 100 mL ethanol were added. The mixture was heated to reflux around ${70}\,^\circ$C and kept for 4 h while vigorously stirring. After cooling, the phases were separated in a separatory funnel, and the crimson organic phase was washed 3 times with ca. 300 mL MQ water. The solvents were evaporated using a rotary evaporator, down to a final vacuum of 40 mbar at ${50}\,^\circ$C which was held for 15 min.

#### Heat-up IONP synthesis

The synthesis followed the general Park method with slight modifications as published previously by our group [28, 35]. A 100 mL round-bottom flask was charged with 25 mL 1-Octadecene and equal amounts of iron oleate (1.6 g = 1.78 mmol assuming Fe($\hbox {OA}^-)_3$) and oleic acid (600 ${\upmu \hbox {L}}$). The charged flask and condenser were flushed with nitrogen for 10 min, then heated at a rate of ${3}\,{\hbox {K min}^{-1}}$ until ${320}\,^\circ$C. The temperature was held for 45 min (dwell time), after which, the reaction was stopped by removing the heating mantle and cooling down the reaction flask with continued stirring. The reaction mixture was transferred from the flask into a beaker using 25 mL *n*-hexane. IONPs were precipitated on a magnet by addition of 100 mL acetone/isopropanol (2:3 ratio). After three washes with acetone, the IONPs were resuspended in 30 mL toluene and stored until further use.

#### Semi-continuous IONP synthesis

The semi-continuous synthesis procedure was adapted from the heat-up synthesis. A 100 mL three-necked round-bottom flask was charged with 25 mL 1-octadecene and variable amounts of oleic acid and connected to a condenser, a thermocouple, and a nitrogen inlet. The setup was flushed with nitrogen for 10 min, thereafter, the inlet was replaced by a rubber septum under $\hbox {N}_2$ counterflow from the top, and finally heated. When the target temperature was reached, the FeOl precursor was added from a single-use 5 mL syringe through a 120$\times$0.8 mm needle with help of a programmable syringe pump (AL-1000, World Precision Instruments, Shanghai, China), see Fig. 2a. The parameters of the *Standard semi-continuous* are: 900 mg = 1 mmol (Fe($\hbox {OA}^-)_3$) iron oleate, 1.5 mmol OA, set 5 ${\hbox {mL h}}^{-1}$ addition speed, ${320}\,^\circ$C, and 30 min dwell time. A full set of the tested reaction condition is given in Table 2. The syringe pump was programmed in ${\hbox {mL h}}^{-1}$, after addition the accurate rate in ${\hbox {g h}^{-1}}$ was calculated using the exact mass of FeOl dispensed found by subtracting the mass of the syringe and needle after addition and from the mass before and timing the addition. The volume difference arising from varied FeOl and OA amounts was corrected for by adjusting the solvent volume to keep the final reaction volume constant at 26.446 mL. Cooldown and cleaning were executed analogously to the procedure of the heat-up synthesis.

**Table 2:** Reaction conditions showing the different semi-continuous and heat-up experiments performed

Reaction condition	n FeOl	Equivalents OA	Set addition speed	Temperature	t dwell
Unit	mmol	(OA:FeOl)	${\hbox {mL h}}^{-1}$	$^\circ$C	min
Heat-up	1.78	1	n.a	320	45
Standard semi-cont	1	1.5	5	320	30
n FeOl	3	1.5	5	320	30
Addition speed	1	1.5	1	320	30
	1	1.5	10	320	30
	1	1.5	Hot inj.$^1$	320	30
OA equivalents	1	0	5	320	30
	1	0.5	5	320	30
	1	3	5	320	30
Dwell time	1	1.5	5	320	60
	1	1.5	5	320	90
Temperature	1	1.5	5	305	30
	1	1.5	5	290	90
Heat-up like	1.78	1	5	320	45

^1^For the *hot injection* variation, the FeOl precursor was manually injected through the needle as fast as possible, within 60 s

#### Phase transfer

The IONPs were transferred from toluene to cyclohexane by precipitation with a mix of acetone, propanol and water on a magnet. The supernatant was poured off and the precipitate washed with acetone three times before redispersing. Then the phase transfer was performed following the protocol of Cai et al., scaled to 30 mg IONPs per reaction [42]. The IONP redispersing in cyclohexane, the PVP-*tert*butanol-mixing were supported by sonication in a waterbath sonicator. The microemulsion oxidation reaction was carried out for 2 h on an oscillation shaker (280 rpm), sonicating for 15 min at halftime. The transferred particles were collected by centrifugation until a pellet formed, then washed. The washing steps are 5 mL ethanol, 5 min at 10,000 g, twice; 4 mL water at pH 4 10 min at 20,000 g, 4 mL water at pH 3 10 min at 20,000 g. After each step, the supernatant was poured off and the pellet redispersed by sonication and vortexing. Finally, the particles were redispersed in 4 mL water and the pH adjusted to neutral.

### Characterization

#### Attenuated total reflection-Fourier transform infrared (ATR-FTIR)

Attenuated total reflection - Fourier transform infrared (ATR-FTIR) analysis was performed by casting a drop of iron oleate or IONPs the samples onto the ATR crystal of a Vertex 80v vacuum FTIR spectrometer (Bruker Corporation, Billerica, MA, USA). Spectra were recorded in vacuum, in a range of 400–${4000}\,\hbox {cm}^{-1}$ using 50 scans at a resolution of ${4}\,\hbox {cm}^{-1}$. The FeOl spectra were manually normalized to the $\nu _{as}$($\hbox {CH}_2$) band at ${2922}\,\hbox {cm}^{-1}$.

#### Electron microscopy

Samples for electron microscopy were prepared by casting an aliquot of diluted sample of IONPs in toluene onto Formvar carbon-coated copper grid (300 mesh, Electron Microscopy Sciences) and leaving the solvent to evaporate. Micrographs were taken on a scanning transmision electron microscope (STEM) (SU9000, Hitachi High-Tech) in Bright-Field TEM mode applying an accelation volatge of 30 kV and 15 A current.

#### Vibrating sample magnetometry (VSM)

Oleic acid coated IONPs were precipitated with an excess of acetone/isopropanol mix and dried for 48 h at ${65}\,^\circ$C, yielding a waxy black material. Phase transferred IONPs were dried (48 h, ${65}\,^\circ$C) directly from aqueous solution. Dried samples were transferred into gelatin gel caps and the magnetic response was measured by a Princeton MicroMag Vibrating sample magnetometer by Princeton Measurements Corp. using an induction field of 10 kOe at room temperature, measuring in 200 Oe increments. The specific magnetic saturation was corrected based on the iron content given by MP-AES after digestion of the sample.

#### IONP digestion and determination of iron content by MP-AES

An Agilent 4210 MP-AES Optical Emission Spectrometer was used to determine iron content by Microwave-assisted plasma atomic emission spectroscopy (MP-AES). Prior to analysis, samples of 0.1 to 0.2 g FeOl or VSM samples including the gel cap were digested in 10 mL nitric acid (65%) using a simple program of 15 min heating to ${210}\,^\circ$C and 15 min hold at ${210}\,^\circ$C in a Speedwave XPERT microwave digestion system (Berghof Products + Instruments LTD, Germany). The digested samples were transferred into 100 mL or 250 mL volumetric flasks and filled with water to the exact volume. Calibration curves were prepared from an Iron ICP standard (1 ${\hbox {g L}^{-1}}$ in 2–5% $\hbox {HNO}_3$, VWR Chemicals, Belgium) by dilution with 2% $\hbox {HNO}_3$ to yield iron concentrations of 1 mg $\hbox {L}^{-1}$ to 100 mg $\hbox {L}^{-1}$.

#### Thermogravimetric analysis (TGA)

5 mg to 10 mg sample of IONPs dried for VSM or FeOl in a crucible was loaded into the instrument (TG 209 F1 Libra, NETZSCH-Gerätebau GmbH, Germany). Then the thermogravimetric analysis (TGA) was performed, heating the sample at 20 $\,{\textrm{K min}}^{-1}$ to ${1000}\,^\circ$C under $\hbox {N}_2$. Transitions were identified using the extrema of the first and second deviation of the $T-m$-curve.

#### X-ray diffraction (XRD)

An aliquot of IONP in toluene stock solution was dried on a single-crystalline silicon holder and covered with a 0.4 ${\upmu m}$ kapton film. The scattering patterns were collected using a Bruker D8 ADVANCE DaVinci with Cu K$\alpha$ radiation ($\lambda ={1.5406}$Å) in a $2\Theta$ range of 20$^{\circ }$–80$^\circ$ with 0.4$^\circ$ step size.

#### Dynamic light scattering (DLS) and $\zeta$-potential

Hydrodynamic size and $\zeta$-potential were measured based on dynamic light scattering (DLS) and electrophoretic mobilty in a Litesizer 500 of Aton Paar GmbH (Graz, Austria) equipped with a dosing system (Metrohm AG, Switzerland) in an Omega-type folded capillary cell. Depending on concentration, the IONP stock samples were diluted 1:1 to 1:40 in a small glass vial and redispersed by vortexing and sonication in water bath witch pipette flush. If needed, the pH was adjusted with diluted solutions of HCl and NaOH. Then the hydrodynamic diameter was measured follow by $\zeta$-potential, both in triplicates.

## Supplementary information

Supporting information containing: summary table of reviewed literature, TGA and FTIR details of iron oleate, summary tables of IONP properties (VSM, STEM), STEM images of semi-cont. IONPs and phase transfer IONPs.

## Supplementary Information

Additional file 1 (EPS 610677 KB)

Additional file 2 (EPS 610706 KB)

Additional file 3 (EPS 610746 KB)

Additional file 4 (EPS 3601 KB)

Additional file 5 (EPS 138 KB)

Additional file 6 (EPS 102 KB)

Additional file 7 (EPS 727 KB)

Additional file 8 (EPS 2657 KB)

Additional file 9 (EPS 98 KB)

Additional file 10 (EPS 21226 KB)

Additional file 11 (EPS 68 KB)

Additional file 12 (EPS 66863 KB)

Additional file 13 (PDF 197 KB)

## Data Availability

All data generated or analyzed during this study are available from the corresponding author on reasonable request.
