# Supplementary material for: Insights into semi-continuous synthesis of iron oxide nanoparticles (IONPs) via thermal decomposition of iron oleate
Source: Discov Nano. 2025 Jan 7;20(1):5. doi: 10.1186/s11671-024-04167-6 (PMC11707164; doi:10.1186/s11671-024-04167-6)
Supplement: Supplementary file 13 — Additional file 13 (PDF 197 KB) [file 11671_2024_4167_MOESM13_ESM.pdf]

Table A1 Previously reported studies on semi-continuous thermal decomposition for iron oxide and ferrite nanoparticles

**Reference**	Shape-Controlled Growth and Shape-Dependent Cation Site Occupancy of Monodisperse Fe_3O_4 Nanoparticles<br>C.-H. Ho, [...] C.-H. Lai, **2011** [12]	Enhanced Nanoparticle Size Control by Extending LaMer's Mechanism<br>E. C. Vreeland, [...] D. L. Huber, **2015** [21]	Thermal Decomposition Synthesis of Iron Oxide Nanoparticles with Diminished Magnetic Dead Layer by Controlled Addition of Oxygen<br>M. Unni, [...] C.Rinaldi, **2017** [14]	**This study**	Extended LaMer Synthesis of Cobalt-Doped Ferrite<br>B.D. Fellows, [...] O. T. Mefford, **2019** [15]	Manganese and cobalt substituted ferrite nanoparticles synthesized via a seed-mediated drip method<br>Z. Yan, [...] O. T. Mefford, **2021** [17]
**NPs**	Magnetite	Magnetite	Magnetite	Fe_xO_y	$CoFe_2O_4$	$Mn_{0.5}Co_{0.5}Fe_2O_4$
**Reagents added**	$Fe(acac)_3$ ($0.2\,mol\,L^{-1}$) in BE	Refined FeOl ($0.22\,mol\,L^{-1}$) in ODE	30 mL FeOl ($0.63\,mol_{Fe}\,L^{-1}$) in 55 mL ODE	FeOl	Refined metal oleates from acac in ODE ($0.22\,mol\,L^{-1}$)	Oleates (Mn/Co+Fe, $0.1\,mol\,L^{-1}$) in ODE
**Addition mode**	$10\,mL\,h^{-1}$: cubes (30 min total); $20\,mL\,h^{-1}$ (or $0.3\,mol\,L^{-1}$): spheres	$3\,mL\,h^{-1}$ml/h, syringe pump, needle	$9\,mL\,h^{-1}$ mL/h syringe pump	$5\,mL\,h^{-1}$ mL/h syringe pump, needle	$3\,mL\,h^{-1}$ mL/h, syringe pump, needle	$10\,mL\,h^{-1}$ for 1 h to seed solution
**Synth. conditions**	290 °C, BE +OA +OAm, 1,2-tetradecanediol, 2 h	350 °C, Docosane, +OA, N_2	350 °C, Docosane, up to 5 h, 350 rpm, N_2/Ar	320 °C, +OA, ODE, N_2	350 °C, +OA, Docosane, N_2	1st seed nucleation from (MnCoFe)acac. 360 °C, +OA, 1,2-hexadecanediol, N_2
**Varied Parameters**	4 c(precursor) , 3 c(capping agents)	time by aliquots	4 gas atmosphere, time	precursor amount, $T, \dot{V}$(FeOl), c(OA), time	time, addition rate	seed/precursor types
**Size**	16.1 ± 0.9 nm cubes, 16.4 ± 1.1 nm polyhedral	9 - 35 nm spheres	26 nm no O_2; 24.1 nm w/ O_2	5.6 - 19.1 nm spheres	10 - 22 nm spheres	19 nm; 17.6 nm spheres
**M_{sat}**	n.a.	$97\,emu\,g_{Fe}^{-1}$ @22.7 nm	$24\,emu\,g_{Fe}^{-1}$ @21.4 nm no O_2; $102\,emu\,g_{Fe}^{-1}$ @22.7 nm w/ O_2	32 - $92\,emu\,g_{Fe}^{-1}$	n.a.	$16\,emu\,g_{metal}^{-1}$
**Findings and characterization**	Cubic: {100} terminated, rhombicuboctahedral: {100}{110}; Facet growth governed by intrinsic surface energy and monomer conc.; Fe^{3+} more (stable) in cubic NP; TEM, SAED, XRD, XMCD, XPS, XAS	Continuous growth ($D \propto t^{\frac{1}{3}}$), size prediction model; T_b varies linearly with particle volume; FTIR, SQUID, XRD, SAR, SAXS	Compare heat-up to semi-cont. (N_2 Ar, Ar+O_2); O_2 essential for magnetism, no O_2 polycrystaline, annealing of particles, phase transfer (PEG); STEM, SQUID, XRD, MPS/MPR, MPI	long dwell time: smaller particles w/ low M_{sat}; high T for desired properties, phase transfer(oxidation); STEM, VSM, MP-AES, FTIR	not optimized, linear growth 50-80 min, SAR oscillates over reaction time: indicates multiple nucleations; FTIR, TEM, SAXS, SAR, Calorimetry, ICP-OES	2 NP types (CoFe +Mn, 19.0 ± 1.9 nm; MnFe + Co, 17.6 ± 3.3 nm) Homogeneous crystal phase in XRD+EDX; TEM, EDX, VSM, ICP

BE= benzyl ether, NP= nanoparticle, OA =oleic acid, OAm= Oleyl amine, ODE = 1-octadecene

Table A1 Continued Previously reported studies on semi-continuous thermal decomposition, incl. polyol synthesis, for iron oxide and ferrite nanoparticles
Table A2 *

**Reference**	Gram scale synthesis of Fe/Fe_xO_y core–shell nanoparticles and their incorporation into matrix-free superparamagnetic nanocomposites<br><br>J. Watt, [...] D. L. Huber, **2018** [13]	Synthesis of Fine-Tuning Highly Magnetic Fe@Fe_xO_y Nanoparticles through Continuous Injection and a Study of Magnetic Hyperthermia<br>S. Famiani, [...] N. T. K. Thanh, **2018** [16]	Insights into the Magnetic Properties of Sub-10 nm Iron Oxide Nanocrystals through the Use of a Continuous Growth Synthesis<br><br>S. R. Cooper, [...] J. E. Hutchison, **2018** [18]	Evolution of Atomic-Level Structure in Sub-10 Nanometer Iron Oxide Nanocrystals: Influence on Cation Occupancy and Growth Rates<br><br>S. R. Cooper, [...] J. E. Hutchison, **2020** [19]	Understanding the Effects of Iron Precursor Ligation and Oxidation State Leads to Improved Synthetic Control for Spinel Iron Oxide Nanocrystals<br><br>L. K. Plummer, [...] J. E. Hutchison, **2020** [20]	Continuous growth phenomenon for direct synthesis of monodisperse water-soluble iron oxide nanoparticles with extraordinarily high relaxivity<br>P. Cheah, [...] Y. Zhao, **2020** [44]
**NPs**	Fe/Fe_xO_y	α-Fe@Fe_xO_y	γ-Fe_2O_3	γ-Fe_2O_3@Fe_3O_4	γ-Fe_2O_3/Fe_3O_4	Magnetite
**Reagents added**	$Fe(CO)_5$ (37 mmol) in 15 mL ODE	$Fe(CO)_5$	$Fe(acac)_2$+OA, boiled; Oleyl-OH (separately)	$Fe(acac)_2$+OA, boiled; Oleyl-OH (separately)	FeOl from $Fe(acetate)_2$ or$Fe(acac)_3$	$Fe(acac)_3$(0.1 $mol_{Fe}\,L^{-1}$) in DEG
**Addition mode**	100 $\mu L\,min^{-1}$, syringe pump; 0.33 $mL\,min^{-1}$, peristaltic pump	0.4 $mL\,h^{-1}$, syringe pump	10 $mL\,h^{-1}$, syringe pump, needle	10 $mL\,h^{-1}$, syringe pump, needle	0.2 $mL\,min^{-1}$ (0.1 $mmol_{Fe}\,min^{-1}$), syringe pump, needle	STEPWISE
**Synth. Conditions**	30 min, 250 °C, ODE, OA + TOP; N_2, 1g and 10g-batches	180 °C, ODE, OAm + hexadecylamonium chloride, max 45 min	POLYOL: 230 °C, Oleyl-OH, 20 min, N_2	POLYOL: 230 °C, Oleyl-OH, 20 min, N_2	POLYOL: 230 °C, Oleyl-OH, 20 min, N_2	POLYOL , 230 °C, DEG, Ar
**Varied Param.**	Scale	amount Fe, rate	precursor amount	precursor amount	precursor type, scale	
**Size**	10g: 13.7 ± 2.5 nm, 1g: 15.2 ± 1.2 nm w/ 2.5 nm shell; spheres	12 - 20 nm spheres	4 - 8 nm spheres	10 nm 1.3 nm shell	8.7 ± 2.7 nm Fe^{III}-rich; 10.1 ± 0.9 nm Fe^{II}-rich var. shapes	4 - 14 nm
**M_{sat}**	96 $emu\,g_{Fe}^{-1}$ @50 K @15 nm; 76 $emu\,g_{Fe}^{-1}$ @50 K in composite	35 - 109 $emu\,g^{-1}$	78 $emu\,g_{Fe}^{-1}$ @3.9 nm; 112 $emu\,g_{Fe}^{-1}$ @8 nm	n.a.	n.a.	77 $emu\,g_{whole}^{-1}$ @5 K; 68 $emu\,g_{whole}^{-1}$ @300 K
**Findings**	Scale-up, repeated nucleation events (revers. agglom. mechanism), ligand exchange, epoxy linked resin 62 wt% IONPs, superparamagnetic at RT, high M_{sat}; TEM, SAXS, VSM, DSC, TGA	V(NP) vs. n(Fe) linear, var. core size w/ const. shell thickness, polycryst. shell causes low M_{sat}, NP oxidize during phase transfer: hollow cores/less core; XRD, TEM, EDS, XRD, HAADF, SQUID-VSM, DLS, SAR	Linear growth w/ addition, single-crystal, crystal strain effect on magnetism, constant shell <0.2 nm, SAXS, TEM, XRD, VSM, NIR	Fe_{Td} vacancies catalyt. promote cont.(lin.) fast growth until ≈8 nm, then slower; small (8-3 nm): high av. Fe oxidation state in crystal; bigger size: higher %-Fe_3O_4+ higher V(unit cell); X-ray total diffraction & PDF, TEM, SAXS	Different anisotropies, tunable by precursor ligand and Fe oxidation state; IONP= 60% Fe_3O_4 + 40% Fe_2O_3; elucidate twinning mechanism; scale up: 18.7 ± 2.5 nm; TEM, XRD, UV/vis, FTIR, NIR SAXS	Directly hydrophilic particles; high crystallinity; living growth mechanism; highest r_2 relaxitivity reported; TEM, XPS, XRD, ζ-Pot, VSM, MRI

DEG= Diethylene glycol, NP= nanoparticle, OA =oleic acid, OAm= Oleyl amine, ODE = 1-octadecene, TOP= tri(octyl)phosphane
